# Understanding foot conditions, morphologies and functions in children: a current review

**DOI:** 10.3389/fbioe.2023.1192524

**Published:** 2023-07-19

**Authors:** Hanhui Jiang, Qichang Mei, Yuan Wang, Junhao He, Enze Shao, Justin Fernandez, Yaodong Gu

**Affiliations:** ^1^ Faculty of Sports Science, Ningbo University, Ningbo, China; ^2^ Research Academy of Grand Health, Ningbo University, Ningbo, China; ^3^ Auckland Bioengineering Institute, The University of Auckland, Auckland, New Zealand; ^4^ Department of Engineering Science, The University of Auckland, Auckland, New Zealand

**Keywords:** children, foot morphology, foot posture, obesity, flatfoot, pes cavus

## Abstract

This study provided a comprehensive updated review of the biological aspects of children foot morphology across different ages, sex, and weight, aiming to reveal the patterns of normal and pathological changes in children feet during growth and development. This review article comprised 25 papers in total that satisfied the screening standards. The aim was to investigate how weight changes, age and sex affect foot type, and gain a deeper understanding of the prevalent foot deformities that occur during children growth. Three different foot morphological conditions were discussed, specifically including the effect of sex and age differences, the effect of weight changes, and abnormal foot morphologies commonly documented during growth. This review found that sex, age, and weight changes would affect foot size, bony structure, foot posture, and plantar pressures during child growth. As a result of this biological nature, the children’s feet generally exhibit neutral and internally rotated foot postures, which frequently lead to abnormal foot morphologies (e.g., flat foot, pronated foot, etc.). In the future, attention shall be paid to the causal factors leading to specific foot morphologies during the growth and development of children. However, sufficient evidence could not be provided due to a relatively short period of investigation and non-uniformed research methodology in the current literature. A more comprehensive and in-depth exploration is recommended to provide scientific evidence for the discovery of children foot development and personalized growth pattern.

## Introduction

The human foot, consisting of a total of 26 bones, is one of the most significant parts of the human body (Mauch et al., 2009) and crucial for locomotion. Foot bones and relevant muscles, ligaments, and tendons played significant roles in preserving the general form and ensured functions under static or dynamic conditions (Mauch et al., 2009). In general, the foot is the first to grow during early childhood (Bosch et al., 2009). According to the growth of the foot stopped first, followed by the long bones (the femur and tibia), and lastly the body (Mauch et al., 2009). Reported that the biological performance of the foot in healthy children varied with age (Manousaki et al., 2019). It was discovered that foot width decreased with growing foot length as a normalization of the foot width to length, and adjusting for proportional variations during foot development (Bruner et al., 2009).

As children grow, the foot morphology varied between sex as well. As reported in several studies (Cheng et al., 1997; El et al., 2006; Bruner et al., 2009; Muller et al., 2012; Waseda et al., 2014; Manousaki et al., 2019), the foot length (FL) of boys increased until at least the period of 15 years old, but girls showed scare increases beyond the period of 13 years old. The navicular height (NH) of the boy’s foot exhibited a gradual increase starting at the at 12, followed by a rapid rise at 13, and eventually reached a plateau at 15. In contrast, females’ NH rose gradually at 10, then quickly at 11, and finally hit a plateau at 16^9^.

Weight fluctuations could affect foot morphology, in addition to other variables, such as age, sex, and height. In many developed nations, child obesity is currently at “epidemic” levels (Racette et al., 2003). For public health services across the world, obesity has become an increasing burden and worry for public health services across the world, which is a condition becoming more common, showing long-term medical and social effects (Wang and Lobstein, 2006). Overweight and obesity, according to the World Health Organization, are abnormal or excessive fat deposits that affect general health. Overweight is defined as a body mass index (BMI) for the age that is one standard deviation over the median of the WHO growth reference criteria for school-age children and adolescents (5–19 years). Obesity (de Onis et al., 2010) was defined as a BMI for the age that was more than two standard deviations over the WHO growth reference criterion median. Being overweight put more strain on the musculoskeletal system as kids get older, which could affect their mobility, level of physical activity, and ability to carry out age-appropriate daily tasks. Obesity could cause musculoskeletal discomfort in various body regions (Krul et al., 2009). In addition to musculoskeletal pain and discomfort, being overweight or obese also led to orthopedic issues in the foot and ankle, knee, hip, and spine (Frey and Zamora, 2007; Stovitz et al., 2008). Additionally, this change significantly raised the chance of fractures, growth issues, and developmental problems (Must and Strauss, 1999; Taylor et al., 2006). Being overweight resulted in improper plantar pressure distribution, foot anatomical changes, and foot balance issues (Fink et al., 2019; Park and Park, 2019). Reduced flexibility from the changed foot anatomy stopped children from running or walking activities (Must and Strauss, 1999; Taylor et al., 2006; Fink et al., 2019; Park and Park, 2019). Additionally, being overweight could affect the plantar arch by affecting the bone and ligament support and causing the medial longitudinal arch to collapse (Buldt et al., 2018). Flatfoot was one of the most often reported problems, according to various studies, that such change in the arch may result in related foot conditions (Sachithanandam and Joseph, 1995; Chen et al., 2013; Telfer and Bigham, 2019). However, the two were not discovered to be associated, indicating that there was no connection between obesity and flat feet, in a later set of investigations on weight change and flat feet (Song-Hua et al., 2017).

Flatfoot in quite common in children, and the prevalence was determined by several variables, and the predisposing factors are not only obesity (Abolarin et al., 2011). The prevalence of flat feet may decrease as individuals age (Rao and Joseph, 1992). People who had pes planus (flatfoot) typically exhibited midfoot pronation or hindfoot valgus. Pes planus is a condition in which the medial longitudinal arch (MLA) collapses with the midfoot touching the ground entirely or almost completely (LeGuern et al., 1997; Pfeiffer et al., 2006). The opposite of flatfoot is a pes cavus (high-arched foot), which would not drop with weight bearing. It is typically a deformity because of muscular imbalance, which may be skeletal or soft tissue, or both combined. The deformity is primarily located in the hindfoot, forefoot (midfoot and forefoot), or a combination of both, with varying degrees of severity. According to Zimon et al. (2011) the Charcot-Marie-Tooth (CMT) disease was responsible for half of the pes cavus (Brewerton et al., 1963). Research investigating the timing and progression of foot and ankle changes in children with CMT, as a genetic condition affecting the peripheral nervous system and worsens over time, revealed that approximately 1 in 2,500 individuals was affected by this condition. The weakening of the distal lower extremity, causing foot drop, sensory loss, lacking tendon reflection, muscular spasms, and inverted foot deformity was the typical symptom of CMT (Skre, 1974; Burns et al., 2009). These symptoms resulted in several functional deficits, such as foot discomfort, ankle instability, tripping, falling, poor balance, and foot pain, which would affect gait performance (Redmond et al., 2008a; Ferrarin et al., 2012; Dars et al., 2018). One major symptom was the arch deformity (LeGuern et al., 1997; Zambito et al., 2008), but not always presenting in children. Clinical study, involving 32 children diagnosed with CMT and ranging in age from 7 months to 15 years, reported that 72% exhibited bilateral high arches, while 13% had flat feet (Ghanem et al., 1996; Wines et al., 2005). The result reflected that arch disorders could affect foot morphology.

However, whether increased weight in children could increase the risk of flatfoot disease and the causation of specific foot morphology is unknown. The answer to this question has been controversial, and this study is aimed to discuss the effects of age, weight, and sex differences on foot morphology and focuses on the patterns of abnormal foot changes. The existing studies are still unable to provide sufficient evidence due to factors such as short study periods and non-uniformed research methodology. Therefore, the goal of this review study is to investigate the influence of sex, change in age and weight, and the causal factors leading to abnormal foot morphology. Knowledge could provide a scientific basis for children’s growth and development and the discovery of individualized growth patterns for clinical diagnosis.

## Materials and methods

The study focused on foot morphology changes in children, to discuss the normal and abnormal foot morphologies, thus summarizing the effect of sex, age, and weight differences on foot morphology, especially changes of flatfoot and high-arch foot in the midfoot. The range of this study was set at 0–18 years old according to the growth cycle of the children foot morphology (Bosch et al., 2009; Bruner et al., 2009; Mauch et al., 2009; Waseda et al., 2014; Manousaki et al., 2019), and foot changes caused by genetic or other diseases were excluded in the study. The review did not include data on human rights violations as contained in the Declaration of Helsinki, so ethics committee approval was not required.

### Search strategy

Researchers used databases, including PubMed, Web of Science, and Google Scholar, to conduct a comprehensive literature search strategy. Since our study focused on changes in foot morphology during child development, each search string had to contain the following key words, ‘Children’s feet’ and ‘Morphology’. Therefore, the following key phrases were retrieved using Boolean search syntax, [(Children’s foot) OR (Children’s feet)] AND [(morphology) OR (shape) OR (foot posture)] AND (age) AND (sex), with 134 documents found; [(Children foot) OR (Children feet)] AND [(morphology) OR (shape) OR (foot posture)] AND (foot (Children foot) OR (Children feet) AND [(morphology) OR (shape) OR (foot posture)] AND [(obesity) OR (overweight)], 112 searches [(Children foot) OR (Children feet)] AND [(morphology) OR (shape) OR (foot posture)] AND [(flat foot) OR (Pes cavus) OR (CMT) OR (Foot Valgus)]. Thus, a total of 207 articles were found. The search was not limited to the publication year. Studies released before January 2023 were included. A total of 453 uncensored duplicates were considered for topics, abstracts, and keywords. All copies were removed using reference software (Endnote) and manually checked by the Investigators (HJ, QM, and YG) before the literature screening.

### Inclusion and exclusion criteria

The screening conditions followed the framework construction method of (Linares-Espinos et al., 2018) and the Preferred Reporting Items for Systematic Evaluation and Meta-Analysis (PRISMA) guidelines (Moher et al., 2009).

Inclusion: (1) the language of study is English; 2) young children, children, and adolescents; 0–1 years of age; sample size >1; 3) studies focus on changes in foot morphology; prospective or retrospective studies involved at least changes in foot biomechanics or biological features; abnormal foot type studies should be about the characteristics of morphological changes and injury risk, biological features, plantar pressure; 4) studies should focus on tracer changes over time.

Exclusions: 1) congenital foot disease; other diseases causing foot changes; 18 years of age or beyond; 2) foot has undergone surgery; 3) studies that (apparently) published duplicate results from the same subject sample as in previous publications obtained from the same group; biological feature values reported in the first study were excluded; 4) non-original articles (e.g., comments or conference articles); non-English articles; review; and 5) did not address foot biological features.

## Research risks

Although defining the age range of children between 0 and 18 years, there were still individual differences in studies and even external factors such as race, region, and environment that may affect foot changes. Table 1.

**TABLE 1:** Inclusion and exclusion criteria.

	Inclusion	Exclusion
Research direction	(1) Studies in the English language; (2) Studies should focus on foot morphological changes; Prospective or retrospective studies at least involve changes in foot biomechanics or biological characteristics. In the study of abnormal foot form, the change characteristics relating injury risk to at least one biological characteristic, or plantar pressure; (3) Studies should focus on the tracking of changes in time	(1) The foot has undergone surgery; (2) Studies (apparently) published duplicate results from the same subject sample, the same results in previous publications obtained from the same group; biological features reported in the first study were excluded; (3) non-original articles (e.g., comments or conference articles); non-English articles; review; (4) no biological features of the foot
Subjects and age	children and adolescents; 0–18 years old; sample size> 1	Congenital foot disease; Other diseases lead to foot changes; Over 18 years old

## Result

### Search results

**FIGURE 1:**
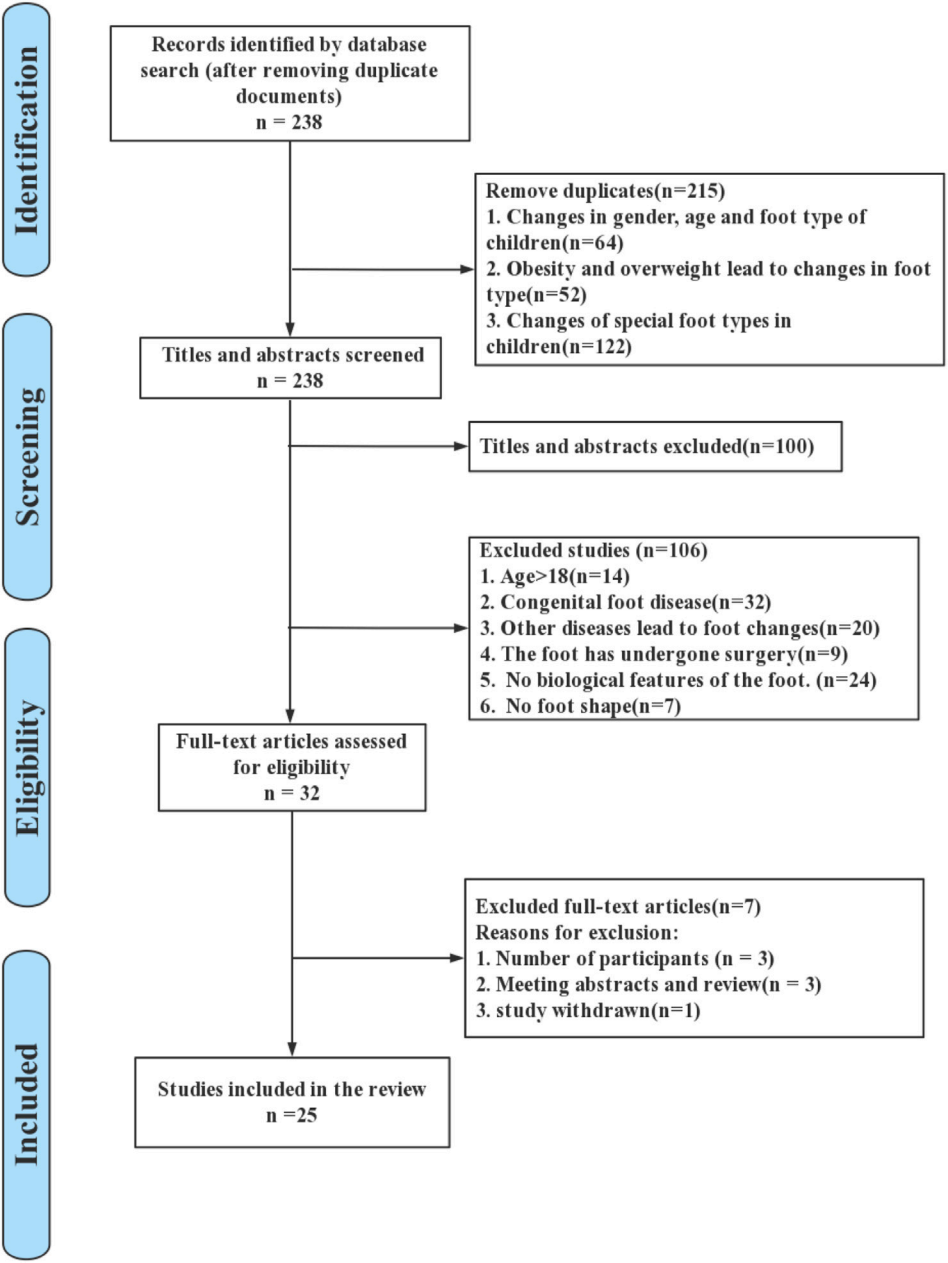
Illustration of literature search and selection process for the current study.

The preliminary search data was 453 articles, and after deleting duplicates, the search yielded 238 articles. Among them, there were 64 articles about sex and age changes in children’s feet. There were 52 articles on changes in the foot due to obesity and overweight. There were 122 articles on changes in specific foot types in children. After removing 100 articles from the keyword filtering in titles and abstracts; 32 studies with congenital foot disorders were excluded; 20 studies with other diseases causing foot changes were excluded; 9 studies with feet undergoing surgical treatment were excluded; 24 studies without foot biology were excluded; and 7 studies without foot morphology were excluded. Therefore, based on the screening eligibility criteria, a total of 32 studies were included for full-text screening. However, 3 studies did not meet the number of participants, 3 conference papers or reviews were excluded, and 1 study was retracted. Finally, 25 papers were found that satisfied the requirements for inclusion. Figure 1 illustrates the systematic search and selection process of the studies in detail.

### Flat foot

The flattened MLA, showing that the foot type had smaller arch angle as well as greater CSI and SI factor volume, was the primary attribute of the flat foot. The typical z-value for the foot length was around −0.1 ^51^.

The findings from Table 2 showed that variations in skeletal architecture, foot posture, and plantar pressure distribution were influenced by differences in sex and age. These variations continued until maturity. Studies on infant foot posture have shown that boys often had flatter feet and lower arches than girls. With increasing age, 46.7% of children aged 8–10 years had neutral feet and 53.3% had medially rotated feet (Al Kaissi et al., 2016). 56.9% of children aged 11 to 13 had neutral feet, 39.7% had medially rotated feet, and 3.4% had posteriorly rotated feet (Al Kaissi et al., 2016).

**TABLE 2:** Influence of Age and Sex on Foot type Changes

References	Participants	Sex	Age	Method	Sex result	Age result
Ana et al. (2017)	2569	1291 girls and 1278 boys	9-15	Researchers compiled data on children's age distribution and mean bilateral FPI-6 scores.	Males typically scored higher than girls did, and the left foot's FPI-6 score was substantially greater than the right foot's (p < 0.05).	The typical FPI-6 score for children and adolescents between the ages of 9 and 15 is 3.0-3.4, with neutral to slightly pronated foot morphology.
Al Kaissi et al. (2016)	150	71 girls and 79 boys	8-13	Determined BMI, weight, FPI and height in the bipedal, static, and relaxed position.	As compared to boys, a somewhat higher percentage of girls had pronated feet.	Children aged 8 to 10 had neutral feet in 46.7 percent of cases and pronated feet in 53.3 percent. 11.9% neutral, 39.7% pronation, and 3.4% pronation were seen in children aged 11 to 13.
Delgado-Abellan et al. (2014)	1031	534 girls and 497 boys	6-12	Measuring the barefoot condition. The boy or girl stood with both feet stilly with equally distributed weight on both feet during measurement.	The aged with the largest sex difference was 8 - 9 years old and 9 - 10 years old. Boys' feet were wider than girls' feet, and the most significant differences between boys and girls of the same age were in ball width, ball circumference, and instep height.	Through all ages evaluated the disparities in foot length increased linearly with height.
Stavlas et al. (2005)	5866	2931 girls and 2935 boys	6-17	The Harris and Beath foot printing mat was used to obtain dynamic (walking) bilateral footprints in every child	At the age of 7, 9, 11, 14 and 15, boys have a significantly higher proportion of low arch than girls of the same age	No systematic description
(Xu et al., 2018)	2543	1303 girls and 1240 boys	7-12	Record foot size through video capture system	At 7-8 and 8–9 years old for girls and 8–9 and 10–11 years old for boys, most measurements dramatically rose. The arch height, instep length and heel width of male and female had the largest increasing trend at the age of 7-12 years (P < 0.05). Most of the sex differences occurred at ages 8, 9, and 11.	In girls 7-8 and 8–9 years old and boys 8–9 and 10–11 years old, the majority of measurements dramatically rose. For both sexes, ages 7 to 12 years, the largest increases were seen in arch height, instep length, and heel width.
Carvalho et al. (2017)	1394	921 girls and 473 boys	10-14	determined weight, height, BMI, and FPI in the bipedal, static, and relaxed position.	With the right foot, boys scored higher than girls.	There were age differences between 11- and 13-year-old for the left foot. The 11-year-old group showed a greater tendency to pronate their feet.
Vrdoljak (2017)	2745	1370 girls and 1375 boys	2-7	Six age categories were used to separate the population of kids. The length of the foot was measured using a measuring tape, while shape was determined clinically.	In all age and sex groups, boys' and girls' left and right feet were identical in length and form.	The second and third years were when the foot grow the fastest. The foot grow by around 1 cm a year from the third to the sixth year.

Remarks: Indicators and scoring standards for the FPI-6 test: (1) Foot posture indexes (FPI-6) Palpation of the talar head; (2) symmetry of the supra- and infra-lateral malleolar curvature; (3) position of the calcaneus in the frontal plane; (4) prominence near the talonavicular joint; (5) congruence of the medial longitudinal arch, and forefoot abduction or adduction on the rearfoot are other examination criteria. The total FPI-6 score ranged from -12 to 12, with each FPI-6 item being graded on a scale of -2 to 2. Based on their FPI-6 scores, the individuals were divided into three groups: 1) 0 to 5 is considered normal; 2) >6 is considered pronated; 3) < -1 is considered supinated (Redmond et al., 2008b).

**FIGURE 2:**
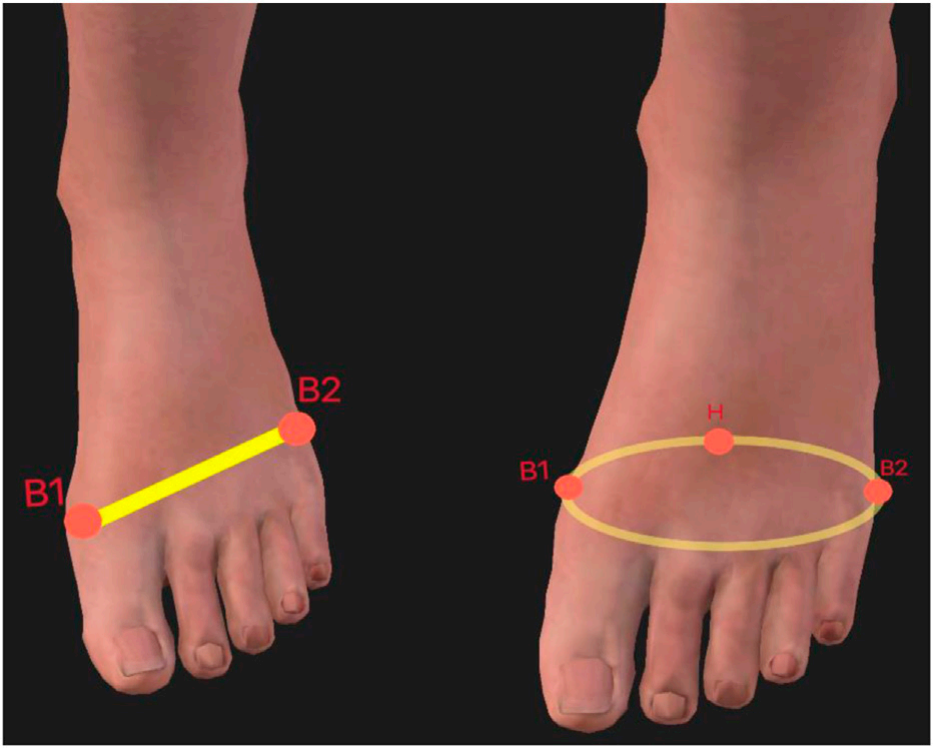
Illustration of Ball width (left) and Ball girth (right).

2. Ball width is the x-y distance between points B1 and B2 projected; The distance between the instep and heel, measured horizontally, is known as the instep distance. Ball girth: the area around the forefoot that corresponds to the B1, B2, and BC points. (B2: fifth metatarsal; B1: first metatarsal; BC points: projected on the z-axis. This is shown in Figure 2.)

From the results in Table 3, (Mauch et al., 2008), concluded that flatfoot occurred at a higher rate in overweight children than in other normal weight and ultralight weight. Although a considerable number of studies have concluded that weight gain during growth can indirectly or directly cause flat feet, (e.g., plantar pressure due to weight gain and flattening of the MLA), the foot prosthesis could maintain the longitudinal arch through compensatory mechanisms (Aboelnasr et al., 2019). As the central nervous system matures in children, individuals would have better motor performance and balance. This would result in better control of lower limb posture (Swallen et al., 2005). Notably, the ossification of foot structures that accompanied skeletal development would allow the arch to remain stable under weight-bearing in children (athirgamanathan et al., 2019). External tibial rotation from the in-toe position at birth to the out-toe position during growth would result in a concomitant decrease in the morphology of the hindfoot exostosis (Atik and Ozyurek, 2014).

**TABLE 3:** Influence of Obesity and Overweight on Foot Types

References	Participants	Age	Category	Method	Result
Agnieszka and Edyta, (2015)	207	4-6	OW	Measurements included body weight, height, body mass index, and Clarke's and gamma angles. Correlations between sex, nutritional health, and variations in foot arch height were also examined.	The proportion of overweight and obese boys and girls increased between the ages of 4 and 6, and those with extra body weight tended to have collapsed medial longitudinal arches of the foot.
Stewart et al. (2018)	3713	3-18	OB	Analysis of pediatric foot dimensions of children's feet (foot length [FL] and foot width [FW])	When compared to their obese counterparts, FL and FW were substantially shorter in male and female patients of normal weight.
Mauch et al. (2008)	2887	2-14	N, UW, OW	Twelve relevant 3D foot measures were taken while the feet were in an upright bipedal position using a 3D foot scanner. The age, sex, height, and weight of the children were also recorded.	Among underweight children, there was a larger percentage of thin feet (65-7%) compared to flat (4-50%), robust (89-100%), and short (21–70%) feet. The disparities were considerably more obvious in the overweight kids. Age-related increases in robustness (69-337%) and flat feet (53-128%) were seen.
da Rocha et al. (2014)	40	6-10	N, OW	Whether individuals were standing on one foot or two feet, the researchers measured the foot sensitivity and plantar pressure and compared the results between the feet and legs of obese and non-obese persons.	Children who are overweight have fewer sensitive feet and more plantar pressure. In addition, sensitivity in various foot areas was comparable in obese and non-obese children.
Gijon-Nogueron et al. (2017)	1798	6-12	OW, OB	Each had their height and weight assessed, and the body mass index (BMI) was computed. The foot posture is described using the foot posture index (FPI).	Body mass does not appear to have a significant impact on static foot posture in children between the ages of 6 and 12.
Jimenez-Ormeno et al. (2013)	1032	6-12	N, OW, OB	The body mass index was determined using measurements of height and weight. Obese, overweight, and normal-weight children were identified. An instatic, three-dimensional foot digitizer was used to measure the foot morphology.	Overweight and obese kids develop bigger feet than their normal-weight peers.
Escalona-Marfil et al. (2022)	575	5-10	N, OW, OB, UW	Foot posture and form evaluation (FPI) and body composition measurement	The findings of this study show that children with normal weight, children who are overweight or obese, as well as children who are underweight, have different foot measurements (FPI, AHI, and MFW).

Remarks: 1. OW=overweight; OB=obeity; N=normal; UW=under weight

From the above analysis, it was clear that flatfoot may not be significantly correlated with changes in body mass index (Hawke et al., 2016; Aboelnasr et al., 2019). In other words, weight gain may not lead to flatfoot. (Hawke et al., 2016). found that children with greater internal rotation of the foot exhibited greater lower limb and overall body flexibility in a study of a sample of healthy asymptomatic children aged 7–15 years. Also, the findings of this study corroborated the discovery of a connection between flat feet and joint flexibility (Pfeiffer et al., 2006).

### High-arch foot

Foot arch deformities were rarely seen in the early childhood population (under 3 years of age). However, as children grow, the navicular bone, the final foot bone to ossify in children between the ages of two and five, was characterized by its fallibility and formative nature. As a result, it became a crucial consideration when evaluating the foot posture of four-year-old kids. Foot navicular ossification occurred later in boys than in girls, while the prevalence of pes cavus increased highly in boys between the period of 4 and 13 years, but the prevalence of pes cavus was frequent in the girl population (Alexander and Johnson, 1989; Aminian and Sangeorzan, 2008; Wozniacka et al., 2013; Chang et al., 2014).

Pes cavus was a common foot disorder in children while this disorder had an overall inversion of the foot, projection of the lateral edges of the foot, and inversion of the heel during standing (Wicart, 2012). This was generally caused by the deformity of pes cavus, which was cavus foot, a simple morphological feature but a normal variant often found in healthy individuals and growing children (Alexander and Johnson, 1989; Schwend and Drennan, 2003; Chang et al., 2014). While the other condition was direct cavus foot, which was the result of foot deformity and often only affected the sagittal plane (forefoot, hindfoot, or both) occurred only in the sagittal plane (forefoot, hindfoot, or both). Direct cavus foot may be associated with multiple causes, and several studies have shown that the condition was due to structural problems in the brain, spinal cord, peripheral nerves, or foot, while neurological disorders were seen primarily in the posterior cavus foot (Schwend and Drennan, 2003; Wicart, 2012). CMT was the most common neurologically caused disorder in this condition (Burns et al., 2009). The risk of deterioration in childhood can generally be averted with the right conservative care (orthotic realignment of the foot) (Wicart, 2012).

## Discussion

### Influence of sex and age differences

Changes in foot morphology in children were gradual over time, particularly the growth of foot length and width. The critical age for foot development was 6 years, and (Waseda et al., 2014). found that children’s foot length increased rapidly from the age of 6 years. Changes in foot morphology characteristics were minimal during the age interval of 10–11 years and stabilized at 12 years (Cheng et al., 1997; El et al., 2006; Catan et al., 2020). Cheng et al. (Cheng et al., 1997) found that children’s foot length and width increased by an average of 8–10 mm per year between the ages of 6–12 years. (Muller et al., 2012). reported that the length and width of the foot grew with age, it was demonstrated that the growth rate of foot length practically hit a plateau at 13 years for girls and 14 years for boys at that point (Evans et al., 2012; Waseda et al., 2014).

In terms of sex differences, the change in the foot was comparable, however, there could be variations between 8 and 10 years old (Delgado-Abellan et al., 2014). According to (Cheng et al., 1997), until the age of 3 years, variations in foot length and width in males were comparable to those in girls. Boys’ feet development increased after the age of 3 years. At the age of 9, boys and girls showed considerable disparities, according to research by Bosch, Gerss, and Rosenbaum (Bosch et al., 2010). Nevertheless, no information regarding foot length was provided in this investigation. (Stavlas et al., 2005). found that the average annual foot length growth rate was 4.3% for boys and 3.9% for girls. The study also revealed that the fastest growth rates were observed at ages 8-9 and 10-11 for boys, and ages 7-8 and 8-9 for girls. Thus, peak growth rates occur earlier in girls than in boys. The age of 7–12 years was the stage of rapid growth in foot arch height among Chinese boys and girls. Studies showed that the navicular height of boys’ feet would increase from 6 to 13 years of age (Szczepanowska-Wolowiec et al., 2021). The navicular or talar navicular joint line was often used to determine the height of medial longitudinal arch. Between the ages of 8 and 13, the arch height of girls rose. The arch height ratio in boys was practically flat until age 11 but considerably rose from age 11 to 13. The formula for the arch height ratio is AHR (%) = navicular height*100/foot length. Girls’ arch height ratios were largely flat until age 10 but considerably rose between age 10 and 12.

Foot morphology changed with growth and development. (Ana et al., 2017). performed the Foot Posture Index (FPI) test for both feet among 2,569 children aged 9–15 years during 2016–2018. The FPI was a clinical diagnostic tool designed to quantify the grade of foot position (posture), such as neutral, internally rotated, or posteriorly rotated. The index was developed using a simple six-factor method to assess foot morphology, to obtain simple and quantitative postures (Al Kaissi et al., 2016). It was reported that girls had a higher percentage of rotated feet than boys, and boys presented higher FPI-6 scores than girls, suggesting that boys had flatter feet and that children and adolescents aged 9–15 years had neutral or mildly internally rotated foot morphology. (Heidi and Priv, 2015). conducted a study on foot morphology in infants who just started walking independently. The study showed that boys’ feet were flatter and had a lower arch than girls’ feet, indicating that males were more likely than girls to have higher FPI-6 scores from an early age.

In other words, the effect of sex differences on foot morphology may be present since birth, and such a condition may exist during growth. The effect of age differences on the foot was also presented from the time of birth, and because of the age difference, the foot width and length grew at different rates, which was a dynamic process. (Al Kaissi et al., 2016). observed differences between children and adult populations, with children generally presenting a neutral foot and internally rotated foot stance, and (Wilkerson and Mason, 2000) found that adults generally presented a neutral foot stance. The study discovered that with increased muscle mass, myelination of motor neurons, and subsequently enhanced muscular strength from puberty forward, the medial longitudinal arch (MLA) had stronger support. This function would contribute to the development of a neutral foot.

In studies of normal foot type, characteristics of foot morphology could be evaluated by footprint, foot index, FPI, manual measurement, and camera acquisition. A large sample of data and studies reported (Alexander and Johnson, 1989; Aminian and Sangeorzan, 2008; Heidi and Priv, 2015; Hawke et al., 2016; Escalona-Marfil et al., 2022) that incorrect gait and walking postures and related external factors were the causes of foot disorders in children.

### Influence of body weight variations

Apart from the above factors of age and sex, variations in body weight and height could also lead to changes in the biological structure of the foot (Waseda et al., 2014; Bailly et al., 2022). Being overweight has implications for foot structure, including alterations of anatomical structure, abnormalities in plantar pressure distribution, and balance (Bruner et al., 2009; Waseda et al., 2014; Manousaki et al., 2019; Bailly et al., 2022). BMI is a popular tool for assessing overweight and obesity as a straightforward measure of the connection between weight and height. The overweight individuals have higher ratios of arch collapse due to thicker layers of adipose tissue (Swallen et al., 2005). Medial section of the plantar foot in a group of 8-year-old children had denser adipose tissue, and the height of longitudinal arch was lower in these observations (Wilkerson and Mason, 2000). (Agnieszka and Edyta, 2015) found that being overweight had a negative effect on the longitudinal arch in preschool children. In preschoolers, no correlation between BMI and the height of the transverse arch was found, but in preschoolers of normal weight, the height of longitudinal arch was significantly increased. In contrast, a trend towards the decreasing longitudinal arch height in overweight and obese boys and girls was found. (da Rocha et al., 2014). concluded that foot sensitivity was lower in obese children than non-obese children. Obese children showed similar sensitivity in all foot regions, while non-obese children were able to differentiate the intensities of touch on different regions. Studies showed that higher sensitivity to touch was associated with higher receptor density, which decreased with age. Therefore, the lower sensitivity in obese children may be due to lower receptor density per surface area unit (Kozłowska, 1988), but this is only theory at this time, and no particular experimental verification of this assumption has been carried out. Future studies may consider examining the receptor density of foot surface area in obese, overweight, normal, and ultralight children as per age group and BMI groups. (Lee et al., 2013).

Overweight boys during childhood may have bigger feet, indicating that obesity may be a major factor affecting foot growth (as determined by the size recorded in the available data set). The structural characteristics of the foot in obese children were wider and thicker, which may increase the peak plantar pressure and vertical peak pressure during gait. Lower footprint angle (FA), higher Chippaux-Smirak index (CSI), higher plantar pressure (Lobstein and Frelut, 2003), higher arch index (AI), and greater footprint area (Stovitz et al., 2008) were found in obese children feet. Children with high body weight may show less degree of variations in the foot morphology or the changes might be gradual or subtle. Thus, (Jimenez-Ormeno et al., 2013), suggested that being overweight may be an important factor affecting foot development in prepubescent students at school. (Mauch et al., 2008). found that low percentage of flatfoot was observed in underweight children. However, the likelihood of flat feet rises with age, although this is highly documented in overweight children.

Obesity would lead to the compensation of foot arch, and either indirectly or directly contribute to the development of flat feet (Sachithanandam and Joseph, 1995; Chen et al., 2013; Song-Hua et al., 2017; Buldt et al., 2018; Telfer and Bigham, 2019). However, a recent study of 728 children between 3 and 15 years old found a relationship between weight and foot morphology (Evans and Karimi, 2015). While Evans (Evans and Karimi, 2015) refuted that heavier children had flatter feet, and further emphasized no association between increased body weight and flatfeet among children. Whilst this statement contradicted with several studies (Sachithanandam and Joseph, 1995; Lobstein and Frelut, 2003; Chen et al., 2013; Telfer and Bigham, 2019), which may be a convergent prediction of the foot morphology in overweight and obese children. Currently, weight reduction was not employed as a treatment in the traditional treatment of flat feet due to obesity. Therefore, it was suggested that further follow-up studies should be conducted focusing on this issue. Yet, alterations in foot morphology brought on by excess weight may cause discomfort or pain and may increase reluctance to exercise, which might result in body weight growth. Considering the development of healthy children, increased attention is still required, especially for physical activity and diet control.

### Abnormal foot type

#### Pes planus (flat foot)

The influence of weight, sex, and age was discussed for the development of flat feet. (Evans et al., 2012). found that between the age of 5–10 years, girls had higher arches than boys. (Ana et al., 2017). investigated 2,569 Japanese kids between the age of 9–15 using the FPI-6, discovering that kids exhibited neutral or moderate internal rotation in standing posture, with a mean score of 3–3.4. Based on a sample of 140 children aged 7–10 years, (Aboelnasr et al., 2019), used FPI-6 screening to identify a sample of 31 kids with flat feet. Basic anthropometric measurements were compared between subjects designated with flat feet, reporting that waist size was associated with foot morphology (although not significantly), but conversely, a “fatter” waist was less associated with flat feet. In other words, weight gain and flat feet were unrelated. In a study by (Hawke et al., 2016), basic data on 30 healthy, asymptomatic kids aged 7 to 15 were gathered, including height and weight (BMI), Beighton score, Foot Posture Index-6 (FPI), and lower extremity evaluation. A correlation between flat feet and joint flexibility was discovered, and internal foot rotation was associated with lower limb and overall body flexibility in healthy and asymptomatic children, but not related with the ankle flexibility (Aboelnasr et al., 2019), which was consistent with a previous statement (Hawke et al., 2016). This finding supported that neutral and internally rotated foot postures predominated over other foot postures, as reported by Evans (Evans et al., 2012) that being overweight would not cause flat feet.

The current review discussed flatfoot based on asymptomatic participants, focusing on the effect of growth and development on the biological shape of the children foot and the potential cause of flatfoot.

#### Pes cavus (high arch)

Cavus foot was used to describe the foot type that had high arch as a typical characteristic. High arch may be caused by a high pitch angle in the hindfoot, hyperflexion of the plantar aspect in the forefoot, or hyperflexion of the midfoot. In complex cases, the cavus foot may be driven by a narrow pitch-heel angle and a possible torsional component in the midfoot. The components of the venous cavity showed increased pronation and pitch of the hindfoot, plantar flexion of the midfoot, and pronation and inversion of the forefoot. The shape of the foot cavity is associated with changes in foot mechanics.

(Alexander and Johnson, 1989) stated that arch deformities were rarely observed in young children (under 3 years) but may occur as children grow. The etiology could be attributed to problems in the brain, spinal cord, peripheral nerves, or foot structure. When motor imbalances occurred before skeletal maturation, the healthy bone morphology may result in substantial changes. When cavernous cavities were acquired after skeletal maturation, there may be little or no change in foot morphology. Two-thirds of adults with symptomatic cavus foot have an underlying neurological condition. CMT disease was the most prevalent. The findings of (Wozniacka et al., 2013) indicated that a high prevalence of high arched feet. Those with symptomatic cavus feet were two-thirds more likely to have a neurological disorder. The most common disease among children and teenagers between the ages of 4 and 13 was the CMT disease. On the right foot, 66.5% of children had high arches, compared to 61.4% on the left, and girls were more likely to have high arches than boys. (Chang et al., 2014). studied static foot morphology, including foot navicular height and arch volume during sitting and standing in 27 children aged 2–6 years. The study found that the arch volume index (AVI) was significantly correlated with pressure changes in the midfoot (Stovitz et al., 2008), which implied that AVI measured in the static position could be correlated with dynamic changes in mid-foot lower-foot loading. However, there was no correlation between AVI and mean pressure and force throughout the stance phase (Evans et al., 2012). As the arch height decreasing, the pressure and force on the medial metatarsal and midfoot plantar increased (Villarroya et al., 2009). Compared with NH, the correlation between arch volume and foot pressure distribution is higher, so the cause of high arch formation may come from changes in foot structure (Putti et al., 2010). Foot structure may alter over time because of weight fluctuations, foot growth, and development, which could eventually cause musculoskeletal disease to manifest.

The studies included in the current review were based on asymptomatic participants, comparing to the different foot types and those asymptomatic individuals who seem healthy but have higher risks of musculoskeletal diseases.

## Conclusion

This review study mainly investigated three issues, including (1) how age and sex variations affect changes in foot morphology, (2) the effect of weight changes on foot morphology, and (3) common abnormal foot morphology during the growth and development of children. Key finding of this review was that sex, age, and weight change would affect foot size, bony structure, foot posture, and plantar pressures during childhood. As per this biological nature, children feet generally exhibit neutral and internally rotated foot postures, which frequently contribute to abnormal foot morphologies (e.g., flat feet and high-arched feet).

This review comprehensively synthesized several published evidence in previous studies, while future studies may consider focusing on the contribution of other factors to specific foot shapes during child development (in addition to the studies presented here) and further expand the information of the research and application of foot morphologies and conditions in children, thus providing comprehensive knowledge for healthy children development.

## Data Availability

The original contributions presented in the study are included in the article/Supplementary material, further inquiries can be directed to the corresponding author.
